# Ultrafast Spectroscopy of Fano-Like Resonance between Optical Phonon and Excitons in CdSe Quantum Dots: Dependence of Coherent Vibrational Wave-Packet Dynamics on Pump Fluence

**DOI:** 10.3390/nano7110371

**Published:** 2017-11-04

**Authors:** Victor Nadtochenko, Nikolay Denisov, Arseniy Aybush, Fedor Gostev, Ivan Shelaev, Andrey Titov, Stanislav Umanskiy, Dmitry Cherepanov

**Affiliations:** 1 N.N. Semenov Institute of Chemical Physics, Russian Academy of Sciences, Kosygina st., 4, Moscow 119991, Russia; aiboosh@gmail.com (A.A.); boatsween@yandex.ru (F.G.); shelaev@bk.ru (I.S.); scorpio60@mail.ru (A.T.); unan43@mail.ru (S.U.); tscherepanov@gmail.com (D.C.); 2 Institute of Problem of Chemical Physics, Russian Academy of Sciences, Chernogolovka 142432, Russia; dnn@icp.ac.ru; 3 Chemical Faculty, Moscow State University, Leninskie Gory, Moscow 119992, Russia

**Keywords:** quantum dots, CdSe, electron-phonon coupling, femtosecond pump-probe, coherent phonons, wave packets, LO phonon, LA phonon, quantum oscillations, Fano-like resonance, continuous wavelet transform, spectrogram

## Abstract

The main goal of the present work is to study the coherent phonon in strongly confined CdSe quantum dots (QDs) under varied pump fluences. The main characteristics of coherent phonons (amplitude, frequency, phase, spectrogram) of CdSe QDs under the red-edge pump of the excitonic band [1S(e)-1S_3/2_(h)] are reported. We demonstrate for the first time that the amplitude of the coherent optical longitudinal-optical (LO) phonon at 6.16 THz excited in CdSe nanoparticles by a femtosecond unchirped pulse shows a non-monotone dependence on the pump fluence. This dependence exhibits the maximum at pump fluence ~0.8 mJ/cm^2^. At the same time, the amplitudes of the longitudinal acoustic (LA) phonon mode at 0.55 THz and of the coherent wave packet of toluene at 15.6, 23.6 THz show a monotonic rise with the increase of pump fluence. The time frequency representation of an oscillating signal corresponding to LO phonons revealed by continuous wavelet transform (CWT) shows a profound destructive quantum interference close to the origin of distinct (optical phonon) and continuum-like (exciton) quasiparticles. The CWT spectrogram demonstrates a nonlinear chirp at short time delays, where the chirp sign depends on the pump pulse fluence. The CWT spectrogram reveals an anharmonic coupling between optical and acoustic phonons.

## 1. Introduction

Quantum dots (QDs), and in particular CdSe QDs, have broad applications in novel electronic and photonic devices due to their unique optical and electronic properties. Specific QDs optical and electronic properties are a result of their strong quantum confinement effect [1,2,3]. One of the unresolved problems in quantum dot physics is the effect of quantum confinement upon exciton-phonon coupling, especially under high intensity of femtosecond pump pulse. The exciton-phonon coupling is very important to quantum dot photonics because it is a crucial parameter for optical, thermodynamic, and electric properties [4,5,6,7,8,9,10,11,12,13,14,15,16,17,18,19,20,21,22,23]. QDs lattice vibrations reveal two types of phonon modes, that are optical and acoustic phonons [13]. The studies on CdSe QDs longitudinal-optical (LO) [5,6,7,8,9,10,11,12,13], surface optical [12], transverse [24], and longitudinal acoustic (LA) phonon modes have been reported [4,5,6,7,8,9,10,11,12,13,14,15,16,17,18,19,20,21,22,23]. In these works, electron-phonon coupling for optical phonons has been attributed to the Fröhlich interaction(s) [13]. The photogeneration of coherent acoustic phonons (wave packets) can be due to the deformation potential mechanism, the thermoelasticity, the inverse piezoelectric effect, and the semiconductor electrostriction [4,5,6,7,8,9,10,11]. The coupling of acoustic modes is caused by the deformation and the piezoelectric potentials. The deformation potential corresponds to the shift in the electronic band energies induced by static displacements of the nuclear positions. The piezoelectric potential occurs as a result of the macroscopic electric polarization caused by an acoustic vibration [4,5,6,7,8]. In ultrafast laser spectroscopy experiments, the pump pulses have enough bandwidth to cover several excitonic and vibronic levels. Such pulses can coherently excite the multipart excitonic structure and induce the wave-packet dynamics in nanocrystals. A femtosecond pump pulse can create a phonon wave packet via coherent superposition of phonon eigenstates [25]. The vibrational coherence can exist as a wave packet in the excited electronic state and is denoted as displacive excitation of coherent phonons (DECP). Otherwise, the first interaction with the pump pulse can create an electronic coherence. The second interaction with the pump pulse generates a vibrational coherence in the ground electronic state. This pathway is designated as a resonance impulsive stimulated Raman scattering (RISRS). Both routes can result in experimentally observable coherent oscillations in the pump/probe signals. The observation of coherent phonon wave packets in CdSe QDs or nanoplatelets has been reported by several groups [5,6,7,8,9,10,11]. Exciton–phonon coupling was tested by chirped femtosecond pump pulse using pump/probe spectroscopy of colloidal CdSe nanocrystals [26]. Quantum dots may furthermore contain multiple excitations within one nanocrystal under high light fluence [27,28,29,30,31]. The creation of multiexcitons may result in a perturbed level structure through many-body Coulomb interactions [28,29,30,31,32]. The formation of a coherent phonon wave packet under such pump conditions is almost not studied. The pump-power dependence of a coherent acoustic phonon in colloidal CdSe/CdS core/shell nanoplatelets has been recently reported [5]. The main goal of the present work is to study the coherent phonon in CdSe quantum dots as a function of the pump fluence. We report the main characteristic of coherent phonons (amplitude, frequency, phase, spectrogram) in strongly confined CdSe QDs under the red-edge pump of the excitonic band [1S(e)-1S_3/2_(h)]. We demonstrate for the first time that the amplitude of the coherent optical phonon in CdSe nanoparticles excited by a femtosecond unchirped pulse shows a non-monotone dependence on the pump fluence. Time frequency representation of the oscillating signal corresponding to LO phonons revealed by continuous wavelet transform (CWT) shows a destructive quantum interference close to the origin of distinct (optical phonon) and continuum-like (exciton) quasiparticles. The CWT spectrogram demonstrates a nonlinear chirp at short time delays, where the chirp sign depends on the pump pulse fluence. 

## 2. Results and Discussion

### 2.1. Pump-Probe Transients vs. Pump Fluence

**Figure 1:**
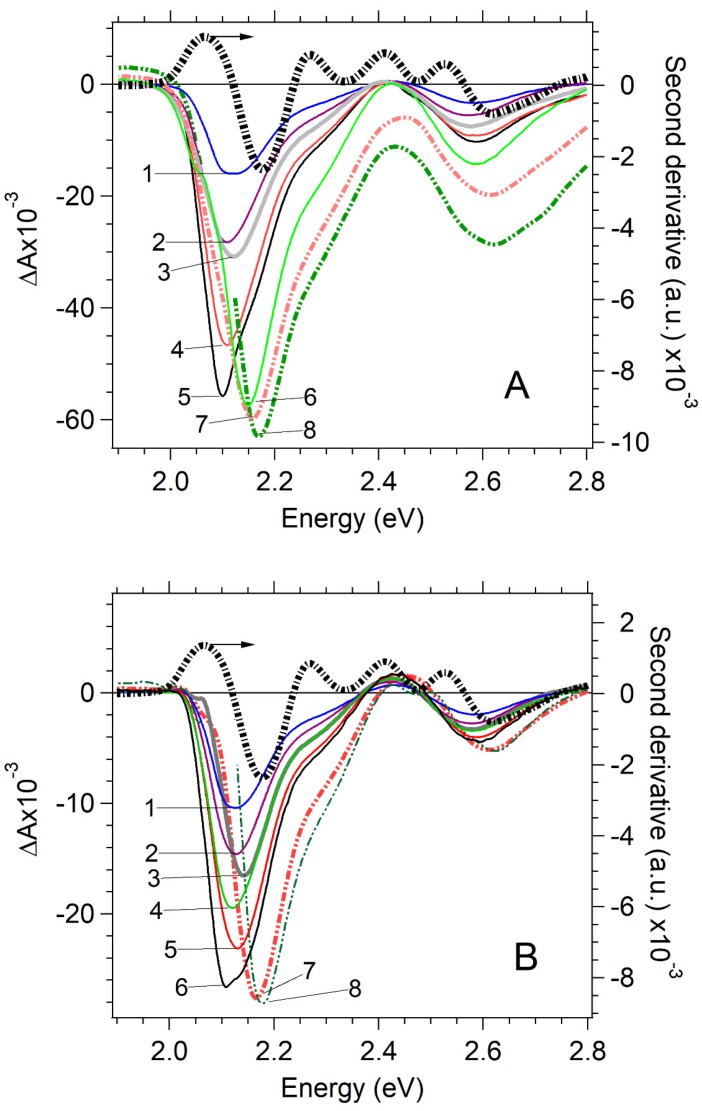
Dependence of transient absorption spectra of CdSe on the pump pulse fluence (left axis) at a short time delay of 100 fs and a long time delay of 500 ps. The second derivative of the CdSe absorption spectrum (black dashed line, right axis). Second derivative minimums demonstrate the position of exciton bands of the absorption spectrum. (**A**) Transient spectra at a time delay of 100 fs: 1, 0.042 mJ/cm^2^; 2, 0.064 mJ/cm^2^; 3, 0.085 mJ/cm^2^; 4, 0.18 mJ/cm2; 5, 0.25 mJ/cm^2^; 6, 0.35 mJ/cm^2^; 7, 1.06 mJ/cm^2^; 8, 1.8 mJ/cm^2^. (**B**) Transient spectra at a time delay of 500 ps: 1, 0.042 mJ/cm^2^; 2, 0.064 mJ/cm^2^; 3, 0.085 mJ/cm^2^; 4, 0.18 mJ/cm^2^; 5, 0.25 mJ/cm^2^; 6, 0.35 mJ/cm^2^; 7, 1.06 mJ/cm2; 8, 1.8 mJ/cm^2^.

**Figure 2:**
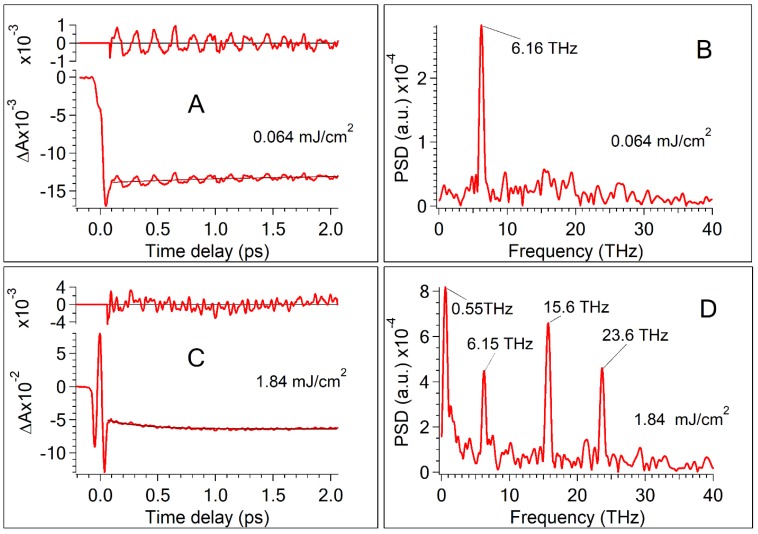
Transient kinetics of CdSe quantum dots (QDs) in toluene at a low and high level of pump fluence. The probe wavelength is 567 nm. Oscillation components were revealed as residuals of model function subtraction from the transient traces. Pump fluence: (**A**) 0.064 mJ/cm^2^; (**C**) 1.84 mJ/cm^2^. Power spectral density (PSD) calculated by Fast Fourier Transform (FFT) of oscillation components. Pump fluence: (**B**) 0.064 mJ/cm^2^; (**D**) 1.84 mJ/cm^2^.

The absorption and photoluminescence spectra of CdSe QDs exhibit the presence of absorption and luminescence exciton bands without a meaningful amplitude of the deep trap luminescence (see SI1, Figure S1). The averaged core size of the particles was 3.7 nm, determined by the position of the lowest excitonic transition [1S(e)-1S_3/2_(h)] at 573 nm (SI1, Figure S1). The 30 fs pulse pump at 590 nm centered at the red-edge of the exciton absorption band can generate excitons at the band edge without an excess of electronic energy for dissipation (SI1, Figure S1). The transient absorption (TA) spectra reveal the bleaching bands (due to state-filling effect), the excited state absorption (due to inter-band transitions), and induced absorption (due to a multiexciton caused by level shiftings or optical transitions involving interface states) [6,7,8,9,10,11,27,28,29,30,31,32,33]. TA spectra corresponding to the different pump pulse energies are shown in Figure 1 for a short time delay of 100 fs and a long-time delay of 500 ps. These spectra manifest main spectral features related to the TA of CdSe. The bleaching bands correspond to transitions [1S(e)-1S_3/2_(h)] at 2.1 eV, [1S(e)-2S_3/2_(h)] at 2.3 eV and [1S(e)-1S_1/2_(h)] at 2.56 eV. The assignment of bleaching bands was made according to [28,29]. Excitonic and multiexcitonic contributions to the transient absorption signal have been extensively discussed previously [6,7,8,9,10,11,27,28,29,30,31,32,33] and will not be discussed further here. Figure 2 shows transient decay curves corresponding to the bleaching at band-edge. In doing so, transient decay traces depend on the pump pulse fluence (all set of data SI2, Figure S2). The dependence of transient decay kinetics on the pump fluence due to the process of the multiexciton recombination and surface trapping was reported previously [1,21,28,29,31,35].

### 2.2. Oscillations in Transient Traces of CdSe QDs Dissolved in Toluene

Figure 2 shows a periodic modulation of the transient absorption signal for two pump fluences (all set of data see SI2, Figure S2) which can be unambiguously assigned to the appearance of coherent wave packets [5,6,7,8,9,10,11]. The sum of exponentials was used as a model function to fit the nonoscillating excitonic part of the transient signal. The model function was subtracted from the transient traces. The oscillation component of the transient traces is even more observable from the residuals (all set of data see SI2, Figure S3). The oscillations at low pump energies are different from oscillations appearing at high pump energies (Figure 2 and Figure S3). To identify the main frequencies, a Fast Fourier Transform (FFT) of transient traces residuals at 2.186 eV (567 nm) has been performed. The FFT reveals one peak near 6.16 THz (205 cm^−1^) when the pump energy is low. Low pump pulse energy ensures that the sample contains mainly single excited and nonexcited QDs. Figure 2 demonstrates that, at high pump energies, additional peaks develop in the FFT of residuals at 567 nm. High pump fluence means that multiexciton QDs are produced in the sample. These additional peaks are near 0.55 THz (18 cm^−1^), 15.6 THz (520 cm^−1^), and 23.6 THz (786 cm^−1^). The frequency mode of 6.16 THz (205 cm^−1^) is in harmony with cw Raman experiments on CdSe clusters which have been found the LO phonon at 205 cm^−1^ [21] and oscillations in transient traces corresponding to the coherent wave packet of the CdSe LO phonon [5,6,7,8,9,10,11,12,13]. Oscillations at 0.55 THz (18 cm^−1^) can be attributed to the coherent wave packet of LA longitudal-acoustic phonons [6,7]. Oscillations in transient traces at 15.6 THz (520 cm^−1^) and 23.6 THz (786 cm^−1^) inherent CdSe QDs sample at high pump energy also appear in the pure toluene (SI3, Figure S4). Frequencies of these oscillations coincide with the frequencies of Raman peaks of toluene at 522 and 787 cm^−1^ [36,37,38]. Since the excitation of wave packets in toluene corresponds to off-resonant excitation, the RISRS mechanism can be suggested to explain the generation of coherent vibrational wave packets in the ground electronic state of toluene [38,39]. Qualitatively, the difference between oscillations in transient traces (Figure 2A,C) at low and high pump fluence can indicate that at low pump energy mainly the coherent LO phonon is dominant, whereas at high pump energy the coherent LO, LA phonon in CdSe, and vibrational coherent wave packets in toluene are excited, so a superposition of different oscillations is manifested in the modulation of transient traces.

### 2.3. Specific Features of Coherent Phonons Produced in CdSe Revealed by FFT

**Figure 3:**
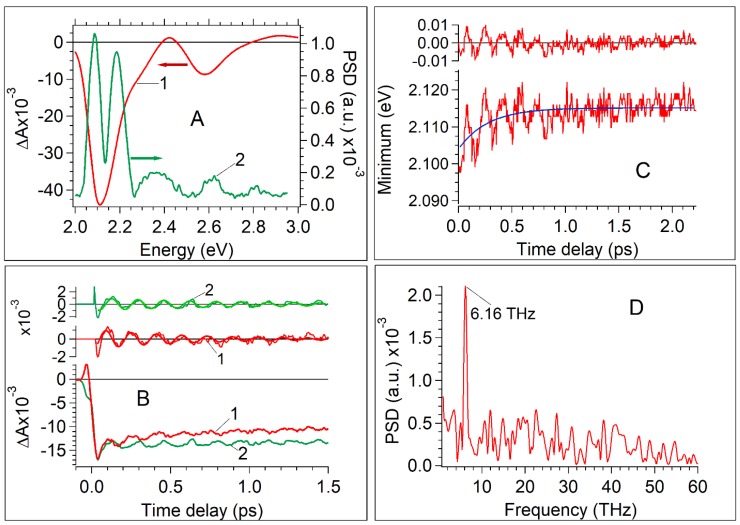
Oscillation components related to the coherent longitudinal-optical (LO) phonon manifestation in pump-probe transients. The data corresponds to the pump fluence of 0.064 mJ/cm^2^. (**A**) 1. Transient spectrum at time delay of 100 fs (red); 2. Amplitude of coherent LO phonon oscillation vs. probe wavelength. (**B**) Phase of coherent LO phonon oscillation: 1. Probe wavelength equals 594 nm (red); 2. Probe wavelength equals 567 nm (green). Oscillation components were revealed as residuals of the exponential fitting of relevant traces. The best fits of oscillation components by the function of exp(−*t*/*τ*_D_)·sin(*ωt* + *ϕ*) are shown by smooth solid lines (see Figure S6). (**C**) Kinetics of the minimum position of the [1S(e)-1S_3/2_(h)] bleaching band. The kinetics of the minimum shift was approximated by the exponential function ~exp(−invTau*t) with a constant of invTau = 3.6 ± 0.4 ps^−1^. The amplitude of the minimum bleaching band shift is close to 7 meV. (**D**) FFT of the oscillation components revealed as a residual shown in (**C**).

Figure 3 shows the main effects associated with the manifestation of oscillations of coherent LO phonon wave packets at low pump energy when oscillations at 15.6 THz and 23.6 THz do not perturb transient traces. A power spectral density (PSD) spectrum calculated by the FFT of residuals at different probe wavelengths shows extremums for coherent LO and LA phonon wave packets. The PSD spectrum of the LA coherent phonon of 0.55 THz manifests a maximum at 594 nm (see the 3D-presentation of PSD (*ν*, *λ*) in SI3, Figure S5). Electron-phonon coupling of LA phonons in bulk and nanocrystal QDs was studied previously [5,6,7,8,9,10,15,18,23,41]. In the present work, LA phonons are not analyzed in detail. Figure 3A demonstrates the PSD (*ν* = 6.16 THz) spectrum of coherent LO phonon oscillations. It manifests two global extremums in the region of the [1S(e)-1S_3/2_(h)] bleaching band at 2.087 eV (594 nm) and 2.186 eV (567 nm). Figure 3B shows oscillations in transient traces at probe wavelength 2.087 eV (594 nm) and 2.186 eV (567 nm). Figure 3B also demonstrates the residuals of these transient traces and their fitting by damped oscillating function A·exp(−*t*/*τ*_damp_)·sin(*ωt* + *ϕ*) according to the *χ*^2^ criteria.

Most commonly, transitions occur between time-dependent electron-vibrational states (electron-vibrational wave packets). The formation of the vibrational wave packets would appear as a frequency modulated dynamic spectrum. Vibrational wave packets can also modulate the dipole transition moment [6]. An amplitude modulation of the dynamic spectrum can be due to the oscillating transition moment. In both cases, transient curves can exhibit oscillations. Mechanism behind the modulations can be distinguished by means of comparison of transients traces recorded at different probe wavelengths [6]. If amplitude modulation occurs, all probe wavelengths would reveal in-phase oscillations. On the contrary, frequency modulation would exhibit phase shifts based upon the probed regions. For instance, oscillations of *ν* = 6.16 THz probed at the red (594 nm) and blue (at 567 nm) edges of the bleaching band (Figure 3A) would be shifted out of phase by π. Dworak et al. [11] reported the phase shifts equal to π for oscillations at different wavelengths. Figure 3B shows that the experimental phase shift only approximately equals π. Moreover, the fitting of residuals by a damped oscillation function gives also approximate results (SI3, Figure S6). It was impossible to obtain a good fit for all time intervals where the oscillations exist, since the frequency providing a good fit for short time delays does not match that at longer time delays. The frequency ω determined from fitting residuals at 567 nm and 594 nm are close in value, but not the same for two probe wavelengths: (1) *λ* = 567 nm, *ω* = 38.15 ± 1.15 × 10^12^ Hz (*ν* = 6.07 THz); (2) *λ* = 594 nm, *ω* = 39.13 ± 0.12 × 10^12^ Hz (*ν* = 6.23 THz) (details about residuals fitting by damped oscillation function are present in SI3, Figure S6). To reach a better approximation for the coherent LA phonon, it was suggested that the linear chirp as a fitting parameter be taken into account [40]. We shall analyze the chirp occurrence below.

Figure 3C demonstrates the spectral shift of the [1S(e)-1S_3/2_(h)] bleaching peak at a short time delay. Exponential approximation of the kinetics of the spectral shift shows that the time constant of the shift is equal to 250 ± 10 fs. The amplitude of the shift is equal to ~10 meV. Oscillations modulate the trace of the bleaching peak shift. The amplitude of the oscillation is close to 10 meV. The Fourier transform reveals a frequency of 6.16 THz coinciding with the LO phonon frequency (Figure 3D) which means that the spectrum of the bleaching band is dynamically modulated by oscillations with the LO phonon frequency. To examine a possibility of the transition dipole moment modulation the kinetic behavior of band integral [41] in a spectral region of interest (*λ*_1_, *λ*_2_) corresponding to red-edge exciton bleaching band was evaluated.

BI(λ1,λ2,t)=1ln(λ2λ1)∫λ1λ2delta Absorbance(λ,t)dλλ

There is no oscillating modulation registered in the kinetics of the band integral (see SI3, Figure S7), whereas the signal modulation is well visible in the transient traces (see Figure 2A and Figure 3B). It suggests that the transition dipole moment modulation is not meaningful for the manifestation of the coherent LO phonon in pump-probe transients of CdSe QDs. Concluding this experimental section, it will be appropriate to suggest that the observed oscillation corresponding to the LO phonon in transient traces relates mainly to the frequency modulated dynamic spectrum of CdSe. It is assumed that coherent phonons influence the electronic states of QDs via the Fröhlich interaction [6,11,13]. The predicted phase shift at different probe wavelengths equal to π is roughly valid, and the fitting of oscillations in residuals of transient traces at different probe wavelengths by the damped oscillation function is valid approximately.

### 2.4. Amplitude of Oscillations Revealed by FFT vs. Pump Fluence

**Figure 4:**
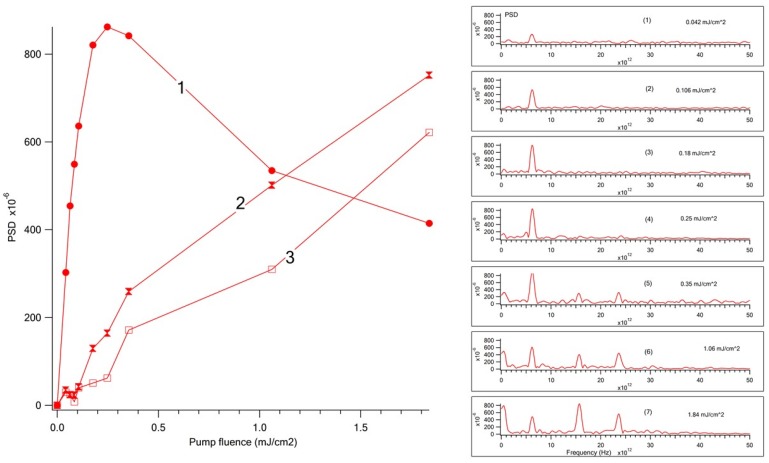
Dependence of the oscillation amplitude in transient traces at 567 nm on the pump fluence. (**Left**) 1. The oscillation amplitude of the coherent LO phonon at 6.16 THz; 2. The oscillation amplitude of the coherent longitudinal acoustic (LA) phonon at 0.55 THz; 3. The oscillation amplitude of the toluene wave packet at 15.6 THz. (**Right**) The dependence of PSD spectra, revealed by the FFT of oscillation components, on the pump fluence: (1) 0.042 mJ/cm^2^; (2) 0.106 mJ/cm^2^, (3) 0.18 mJ/cm^2^, (4) 0.25 mJ/cm^2^, (5) 0.35 mJ/cm^2^, (6) 1.06 mJ/cm^2^, (7) 1.84 mJ/cm^2^.

The significant difference between the oscillating curves obtained at low and high pump fluence (Figure 2B,D) suggests that the excitation of the LA phonon modes at 0.55 THz and the induction of coherent wave-packet dynamics of toluene at 15.6, 23.6 THz develop much steeper with increasing pump fluence than the amplitude of the LO phonon oscillations at 6.16 THz enhances. Figure 4 shows the dependence of PSD amplitude and Fourier transform spectra on the pump fluence. The oscillation of the coherent LO phonon dominates in the FFT spectrum at low pump fluences, while at high pump fluences, the oscillation amplitudes of the coherent LA phonon and toluene modes outstrip the oscillation magnitude of the coherent LO phonon. It is noteworthy that the dependence of the oscillation amplitude of the coherent LO phonon is non-monotonic vs. pump fluence (Figure 4). At low pump fluences, the amplitude of the oscillations increases, reaches a maximum, and at high pump fluences a decrease of the LO phonon amplitude is registered. The amplitudes of competitive modes (the coherent LA phonon at 0.55 THz and coherent wave packets in toluene at 15.6 THz and 23.6 THz) rise monotonically with increasing pump fluence. The linear growth of the coherent wave-packet amplitude of toluene is in accord with theoretical predictions and experimental observations [38,39]. The dependence of the coherent LO phonon amplitude vs. pump fluence shown in Figure 3A was unexpected for us, because this dependence exhibits the maximum. The wavelet transform of LO phonon oscillations reveals striking details of the spectral and temporal structures of the LO phonon at different pump fluences and allows a deeper understanding of the electron-phonon coupling in QDs.

### 2.5. Analysis of the CWT Spectrogram Obtained for Varied Pump Fluence

**Figure 5:**
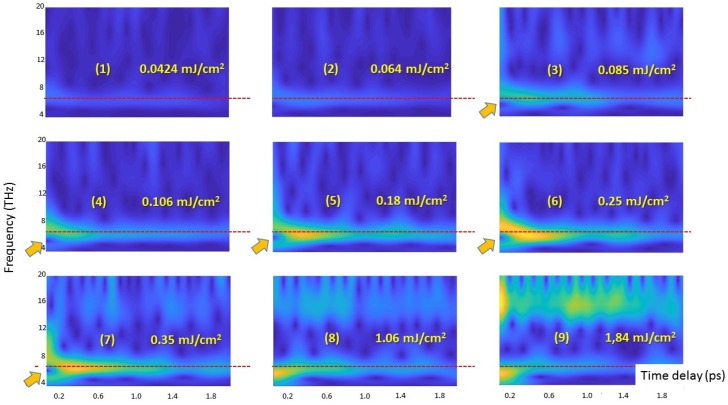
Oscillation intensity distribution by continuous wavelet transform (CWT) in the frequency time domain. Transient traces at the probe wavelength of 567 nm were analyzed. The red dashed line indicates the position of the LO phonon at 6.15 THz as revealed by the Fast Fourier Transform (FFT). Arrows indicate the destructive interference spot. The colormap is the same for all 2D plots revealing the dependence of CWT intensity on pump fluence. Pump fluence: (1) 0.0424 mJ/cm^2^; (2) 0.064 mJ/cm^2^; (3) 0.085 mJ/cm^2^; (4) 0.106 mJ/cm^2^; (5) 0.18 mJ/cm^2^; (6) 0.25 mJ/cm^2^; (7) 0.35 mJ/cm^2^; (8) 1.06 mJ/cm^2^; (9) 1.84 mJ/cm^2^.

A further tool available for analyzing our data is the CWT, which yields instantaneous frequencies and amplitudes of the coherent phonon vibrations. CWT is very helpful for the identification of damping in dynamic systems. CWT is also quite resistant to noise in the signal [42,43]. Figure 5 shows CWT 2D plots corresponding to the frequency region of the LO phonon calculated for the transient trace at 567 nm. Figure 5 demonstrates that the temporal evolution of LO phonon oscillation depends on the pump fluence. The oscillation frequency is chirped. The main changes of the oscillation frequency occur in the time window shorter than ~300 fs. The spectral shift of the bleaching band occurs in the same time window. At low pump fluences the chirp sign defined as the difference between the frequency at initial time delay and the frequency of the LO phonon (6.16 THz) is negative, that is, a softening of the oscillation frequency is registered (Figure 5 and SI4, Figure S8). Increasing fluence enlarges initially only the chirp amplitude, but when the fluence exceeds a well-defined threshold of 0.8 mJ/cm^2^, the chirp changes its sign to positive (Figure 5, SI4, Figures S8 and S9). Figure 4 depicts a matching between the fluence intensity where the amplitude of the coherent LO phonon begins to decrease (≥0.5 mJ/cm^2^) and the pump fluence threshold corresponding to the change of the chirp sign. The decrease of the coherent LO phonon amplitude correlates with the pump fluences corresponding to the negative chirp. The synchronized change of the chirp sign and the change in the dependence character of the amplitude on pump fluence suggest meaningful alternations in the mechanism of the coherent LO phonon origin under increasing pump fluences. The chirp of the coherent LA phonon was documented in the bulk CdSe [40]. To obtain a better fit of the observed oscillations by damped oscillatory functions, Wu, W. and Wang, Y. took the chirp into account [40]. Complex changes in amplitude and frequency of the coherent LO phonon in bulk Bi have also been reported [44,45]. The authors suggested that the manifestation of complex dynamics of observed oscillations associated with the continuum responsible for the interference includes both the electronic and lattice degrees of freedom. It was demonstrated that, under intense optical excitation, the frequency of the fully symmetric phonon in bulk Bi depends on the time delay on a picosecond timescale. The authors suggested that the phonon has a positive chirp arising either from lattice anharmonicity or from time dependence of the electron–hole plasma density [44,45].

**Figure 6:**
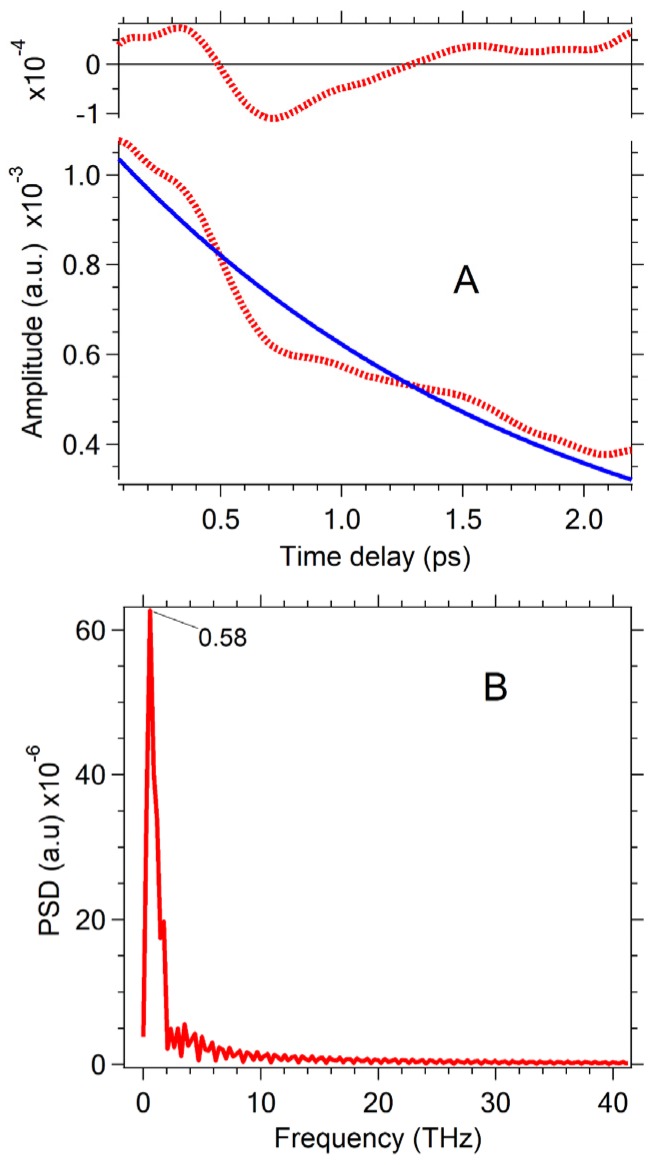
(**A**) Decay of the CWT amplitude corresponding to the coherent LO phonon. Pump fluence is 0.106 mJ/cm^2^. The blue solid line indicates the exponential fit: exp(−*t*/*τ*_CWT_) The time decay *τ*_CWT_ is equal to 1.8 ± 0.2 ps. (**B**) FFT of residuals shown in Figure 6A.

**Figure 7:**
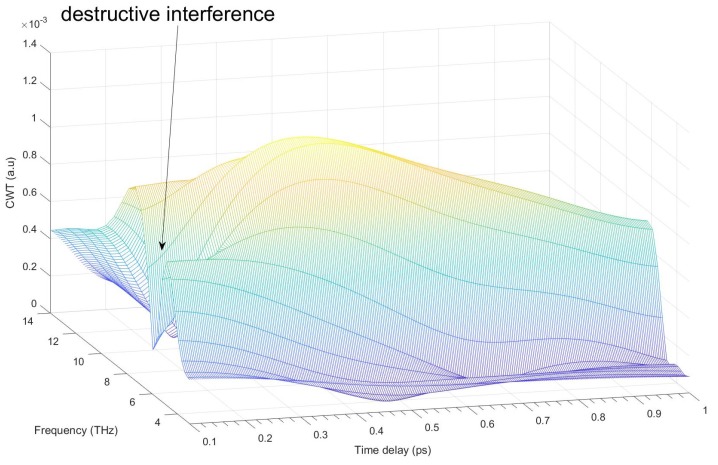
Destructive interference. Spectrogram calculated by CWT corresponding to the transient trace at 567 nm measured at the pump fluence of 0.18 mJ/cm^2^. The 3D plot shows a frequency region around the oscillation of the coherent LO phonon.

Since the oscillation frequency of the LO phonon changes in time (Figure 5), we determine the kinetic curve of the LO phonon amplitude as the CWT ridge value for each time delay in the 3D spectrogram presentation (SI4, Figure S10). The damping rate constant was estimated as exponential fitting of the LO phonon amplitude decay as it is shown in Figure 6 (all set of data see SI4, Figure S10). Increasing fluence leads to the growth of the oscillation damping rate constant (see SI4, Figures S10 and S11). Figure 6 demonstrates the modulation of the kinetic curve of the LO phonon amplitude. It is noteworthy that the FFT analysis of the residual of the exponential decay of this curve reveals that the frequency of this modulation coincides with the frequency of the LA phonon. It suggests the anharmonic coupling between LO and LA phonons. A similar anharmonic coupling of vibrational wave packets for the radial breathing mode (RBM) and the G mode in a carbon nanotube resulting in a frequency modulation of the G mode by the RBM was reported previously in [46]. Figure 5 and Figure 7 show that the coherent response of CdSe reveals an antiresonance dip (destructive interference) at ~ 6 THz in the region of an overlap between the coherent phonon and electronic responses. It assumes a coherent excitation of the coupled phonon-carrier systems and a dynamical interference between short-lived Rabi oscillations and long-lived coherent phonons in CdSe QDs resulting from the coupling between the two oscillations. It should be noted that the excitonic level corresponding to the transition [1S(e)-1S_3/2_(h)] has a fine electronic structure [47,48,49] that reveals the existence of several close electronic levels and not of a continuum spectrum as it is supposed in the Fano resonance theory [50]. We suggest that the interference between electronic and phonon systems can be considered as Fano-like resonance [51]. It was reported that the Fano interference [44,45,52,53] always starts from the destructive one when the corresponding optical phonon being coherent [52,53]. Figure 5 and Figure 7 confirm this conclusion. A similar effect of the Fano destructive interference was reported for bulk silicon [52]. 

It should be pointed out that the amplitude of the oscillations reaches a maximum at the same pump fluence value as that of the sign change of the chirp (~0.8 mJ/cm^2^ Figure 4 and Figure 5 and Figures S8 and S9). The intensity of the coherent LO phonon of CdSe is enhanced by increasing that of the Rabi oscillation due to the enhancement of pump fluence, which indicates that the coherent phonon is driven by the Rabi oscillation through the Fröhlich interaction. The interference picture changes when the critical pump fluence is reached, which is manifested in a qualitative change in the spectrogram at the initial time delay, the chirp changes its sign and, at higher pump fluences, the amplitude of the coherent oscillations of the optical phonon falls.

## 3. Materials and Methods

### 3.1. Femtosecond Laser Photolysis Setup

The femtosecond pump to supercontinuum probe setup was used to measure TA spectra. The output of a Ti:sapphire oscillator (800 nm, 80 MHz, 80 fs, Tsunami, Spectra-Physics, Santa Clara, CA, USA) was amplified by a regenerative amplifier system (Spitfire, Spectra-Physics, USA). The repetition rate was 1 KHz. A pump was carried out by the gauss pulse of 30 fs at 590 nm. H_2_O was used for generation of a supercontinuum. The pump and probe light spots had the diameters of 300 and 120 µm, respectively. The relative polarization of pump and probe pulses was oriented at 54.7° (magic angle). Details of the setup were published previously [54,55].

CdSe QDs were dispersed in toluene. The experiments were carried out at 278 K. The pump pulse operation frequency was 60 Hz. The sample in 200 μm cell was refreshed between incident laser pulses by a pump. Transient spectra of absorbance changes ∆A (*t*, *λ*) were recorded over the range of 380–800 nm. The measured spectra were corrected for group delay dispersion of the supercontinuum using the procedure described previously in [54,55]. The time window of the “coherence spike” seen during the pump-probe overlap was neglected.

### 3.2. Materials

The analytical-grade reagents (Sigma-Aldrich is now Merck KGaA, Darmstadt, Germany) were used without further purification.

### 3.3. Synthesis of CdSe Nanocrystals

The synthesis of CdSe particles is described as follows [56] with some modifications. Briefly, a stock solution of Se precursor was prepared by combining 0.79 g of Se, 8.65 mL of 1-octadecene and 5.2 mL of trioctylphosphine in a 20-mL flask. The mixture was magnetic stirred in an argon atmosphere under warming until Se was dissolved. The Cd precursor was prepared by adding 0.128 g of CdO, 1.26 mL of oleic acid and 12.7 mL of octadecene to a 50-mL round-bottom flask. The mixture was heated at 230 °C with magnetic stirring in argon to dissolve the black powder of CdO, which took about 0.5 h. The additional reagents—trioctylphosphine oxide 2.5 g and pentadecane 6 mL from the previous stage—were consistently added to CdO solution. Then Se precursor solution was injected into the mixture at 230 °C with magnetic stirring in an argon atmosphere. The timing should begin when the selenium solution is added. Samples of 6 mL were taken from the reaction solution at a time interval of 2 min. The products were washed three times with ethanol, followed by centrifugation. Waxy precipitates were obtained which were redispersed in toluene.

## 4. Conclusions

Transient absorption data of CdSe QDs (diameter of 3.7 nm) in toluene was obtained by the broadband femtosecond pump probe technique as a function of the pump fluence. The pump was carried out by 30 fs pulse at 590 nm, at the red edge of the [1S(e)-1S_3/2_(h)] QDs absorption band.

Transient traces demonstrate the oscillation components. The FFT analysis reveals that under low pump fluence oscillations can be assigned to the coherent LO phonon at 6.16 THz (205 cm^−1^). At high pump fluence, coherent LA phonon oscillations at 0.55 THz (~18 cm^−1^) and the coherent wave packet of toluene at 15.6 THz (~520 cm^−1^), 23.6 THz (~787 cm^−1^) dominate. The amplitudes of the LA phonon modes at 0.55 THz and of the coherent wave packet of toluene at 15.6 THz, 23.6 THz enhance more rapidly with increasing pump fluence than the amplitude of the LO phonon oscillations at 6.16 THz.

The dependence of the LO phonon oscillation phase on the probe wavelength suggests that electron phonon coupling is due to the Fröhlich interaction. The amplitude of LO phonon oscillations manifests a non-monotonic dependence on pump fluence, peaking at the pump fluence close to 0.8 mJ/cm^2^. Amplitudes of LA phonon oscillations and toluene wave-packet oscillations show a monotonic growth with increasing pump fluence.

CWT spectrograms evidence that:

(1) Coherent LO phonon oscillations are significantly chirped. An especially significant chirp manifests itself at time delays shorter than ~300 fs. The chirp sign is changed when pump fluence approaches the value of ~0.8 mJ/cm^2^. This fluence threshold matches the fluence where the amplitude of LO phonon oscillations reaches the maximum value.
(2) The coherent response of CdSe exhibits the antiresonance dip (destructive interference) at ~6 THz in the overlap region between the coherent phonon and electronic responses. The obtained data suggests that: (a) this effect is related to the coherent excitation of the coupled phonons and charge carriers; (b) the dynamical interference between short-lived Rabi oscillations is resulting from close electronic states (fine structure) of the exciton and long-lived coherent phonons in CdSe QDs. This data agrees with the conclusion that the Fano interference always starts from the destructive one when the corresponding optical phonon being coherent [47].
(3) The damping rate enhances with the increase of pump fluence.
(4) The anharmonic coupling occurs between optical and acoustic phonons.

## Supplementary Materials

The following are available online at www.mdpi.com/2079-4991/7/11/371/s1, SI1 Absorption and luminescence spectra of CdSe nanoparticles. SI2 Transient decay at different pump fluences. SI3 Fast Fourier Transform. SI4 CWT of transients at 567 nm.

## References

[1] Pietryga J.M., Park Y.S. (2016). Spectroscopic and device aspects of nanocrystal quantum dots. Chem. Rev..

[2] Boles M.A., Engel M. (2016). Self-assembly of colloidal nanocrystals: From intricate structures to functional materials. Chem. Rev..

[3] Jing L., Kershaw S.V. (2016). Aqueous based semiconductor nanocrystals. Chem. Rev..

[4] Ruello P., Gusev V.E. (2015). Physical mechanisms of coherent acoustic phonons generation by ultrafast laser action. Ultrasonics.

[5] Dong S., Lian J. (2017). Pump-Power Dependence of Coherent Acoustic Phonon Frequencies in Colloidal CdSe/CdS Core/Shell Nanoplatelets. Nano Lett..

[6] Sagar D.M., Cooney R.R. (2008). Size dependent, state-resolved studies of exciton-phonon couplings in strongly confined semiconductor quantum dots. Phys. Rev. B.

[7] Sagar D.M., Cooney R.R. (2008). State-Resolved Exciton-Phonon Couplings in CdSe Semiconductor Quantum Dots. J. Phys. Chem. C.

[8] Tyagi P., Cooney R.R. (2010). Controlling piezoelectric response in semiconductor quantum dots via impulsive charge localization. Nano Lett..

[9] Kambhampati P. (2011). Hot exciton relaxation dynamics in semiconductor quantum dots: Radiationless transitions on the nanoscale. J. Phys. Chem. C.

[10] Kambhampati P. (2010). Unraveling the structure and dynamics of excitons in semiconductor quantum dots. Acc. Chem. Res..

[11] Dworak L., Matylitsky V.V. (2011). Coherent longitudinal-optical ground-state phonon in CdSe quantum dots triggered by ultrafast charge migration. Phys. Rev. Lett..

[12] Lin C., Kelley D.F. (2014). The “Surface Optical” Phonon in CdSe Nanocrystals. ACS Nano.

[13] Kelley A.M. (2010). Electron–phonon coupling in CdSe nanocrystals. J. Phys. Chem. Lett..

[14] Kelley A.M. (2011). Electron–Phonon Coupling in CdSe Nanocrystals from an Atomistic Phonon Model. ACS Nano.

[15] Werschler F., Hinz C. (2016). Coupling of Excitons and Discrete Acoustic Phonons in Vibrationally Isolated Quantum Emitters. Nano Lett..

[16] Wise F.W. (2000). Lead salt quantum dots: The limit of strong quantum confinement. Acc. Chem. Res..

[17] Mittleman D.M., Schoenlein R.W. (1994). Quantum size dependence of femtosecond electronic dephasing and vibrational dynamics in CdSe nanocrystals. Phys. Rev. B.

[18] Krauss T.D., Wise F.W. (1997). Coherent acoustic phonons in a semiconductor quantum dot. Phys. Rev. Lett..

[19] Krauss T.D., Wise F.W. (1997). Raman-scattering study of exciton-phonon coupling in PbS nanocrystals. Phys. Rev. B.

[20] Shiang J.J., Risbud S.H. (1993). Resonance Raman studies of the ground and lowest electronic excited state in CdS nanocrystals. J. Chem. Phys..

[21] Alivisatos A.P., Harris T.D. (1989). Electron–vibration coupling in semiconductor clusters studied by resonance Raman spectroscopy. J. Chem. Phys..

[22] Takagahara T. (1993). Electron-phonon interactions and excitonic dephasing in semiconductor nanocrystals. Phys. Rev. Lett..

[23] Cerullo G., De Silvestri S. (1999). Size-dependent dynamics of coherent acoustic phonons in nanocrystal quantum dots. Phys. Rev. B.

[24] Klein M.C., Hache F. (1990). Size dependence of electron-phonon coupling in semiconductor nanospheres: The case of CdSe. Phys. Rev. B.

[25] Mukamel S. (1995). Principles of Nonlinear Optical Spectroscopy.

[26] Mooney J., Saari J.I. (2013). Control of Phonons in Semiconductor Nanocrystals via Femtosecond Pulse Chirp-Influenced Wavepacket Dynamics and Polarization. J. Phys. Chem. B.

[27] Burda C., Link S. (1999). New transient absorption observed in the spectrum of colloidal CdSe nanoparticles pumped with high-power femtosecond pulses. J. Phys. Chem. B.

[28] Klimov V.I. (2007). Spectral and dynamical properties of multiexcitons in semiconductor nanocrystals. Annu. Rev. Phys. Chem..

[29] Klimov V.I. (2000). Optical nonlinearities and ultrafast carrier dynamics in semiconductor nanocrystals. J. Phys. Chem. B.

[30] Klimov V.I., Mikhailovsky A.A. (2000). Quantization of multiparticle Auger rates in semiconductor quantum dots. Science.

[31] Kambhampati P. (2012). Multiexcitons in semiconductor nanocrystals: A platform for optoelectronics at high carrier concentration. J. Phys. Chem. Lett..

[32] Woggon U., Kalt H., Hetterich M. (2004). Spectroscopy of biexcitons and trions in II–VI quantum dots. Optics of Semiconductors and Their Nanostructures.

[33] Cooney R.R., Sewall S.L. (2007). Unified picture of electron and hole relaxation pathways in semiconductor quantum dots. Phys. Rev. B.

[34] Saari J.I., Dias E.A. (2012). Ultrafast Electron Trapping at the Surface of Semiconductor Nanocrystals: Excitonic and Biexcitonic Processes. J. Phys. Chem. B.

[35] Walsh B.R., Saari J.I. (2016). Surface and interface effects on non-radiative exciton recombination and relaxation dynamics in CdSe/Cd, ZnS nanocrystals. Chem. Phys..

[36] Standard Spectra FT-Raman and Scanning (Spex 1403) Data Sets for 50/50 (*v*/*v*) Toluene/Acetonitrile. http://www.chem.ualberta.ca/~mccreery/ramanmaterials.html#toluene.

[37] Polack T., Oron D. (2005). Control and measurement of a non-resonant Raman wavepacket using a single ultrashort pulse. Chem. Phys..

[38] Gdor I., Ghosh T. (2017). Nonresonant Raman Effects on Femtosecond Pump–Probe with Chirped White Light: Challenges and Opportunities. J. Phys. Chem. Lett..

[39] Kumar A.T.N., Rosca F. (2001). Investigations of ultrafast nuclear response induced by resonant and nonresonant laser pulses. J. Chem. Phys..

[40] Wu W., Wang Y. (2015). Ultrafast carrier dynamics and coherent acoustic phonons in bulk CdSe. Opt. Lett..

[41] Kovalenko S.A., Schanz R. (2001). Cooling dynamics of an optically excited molecular probe in solution from femtosecond broadband transient absorption spectroscopy. J. Chem. Phys..

[42] Slavič J., Simonovski I. (2003). Damping identification using a continuous wavelet transform: Application to real data. J. Sound Vib..

[43] Rioul O., Duhamel P. (1992). Fast algorithms for discrete and cntinuous wavelet transforms. IEEE Trans. Inf. Theory.

[44] Misochko O.V., Ishioka K. (2007). Fano interference for large-amplitude coherent phonons in bismuth. J. Phys. Condens. Matter.

[45] Misochko O.V., Ishioka K. (2006). Fully symmetric and doubly degenerate coherent phonons in semimetals at low temperature and high excitation: Similarities and differences. J. Phys. Condens. Matter.

[46] Gambetta A., Manzoni C. (2006). Real-time observation of nonlinear coherent phonon dynamics in single-walled carbon nanotubes. Nat. Phys..

[47] Norris D.J., Efros A.L., Rosen M., Bawendi M.G. (1996). Size dependence of exciton fine structure in CdSe quantum dots. Phys. Rev. B.

[48] Efros A.L., Rosen M. (1996). Band-edge exciton in quantum dots of semiconductors with a degenerate valence band: Dark and bright exciton states. Phys. Rev. B.

[49] Franceschetti A., Fu H., Wang L.-W. (1999). Many-body pseudopotential theory of excitons in InP and CdSe quantum dots. Phys. Rev. B.

[50] Fano U. (1961). Effects of configuration interaction on intensities and phase shifts. Phys. Rev..

[51] Yoshino S., Oohata G. (2015). Dynamical Fano-like interference between Rabi oscillations and coherent phonons in a semiconductor microcavity system. Phys. Rev. Lett..

[52] Hase M., Kitajima M. (2003). The birth of a quasiparticle in Si observed in time-frequency space. Nature.

[53] Lee J.D., Inoue J. (2006). Ultrafast Fano resonance between optical phonons and electron-hole pairs at the onset of quasiparticle generation in a semiconductor. Phys. Rev. Lett..

[54] Shelaev I.V., Gostev F.E. (2008). Primary light-energy conversion in tetrameric chlorophyll structure of photosystem II and bacterial reaction centers: II. Femto-and picosecond charge separation in PSII D1/D2/Cyt b559 complex. Photosynth. Res..

[55] Cherepanov D.A., Shelaev I.V. (2017). Mechanism of Adiabatic Primary Electron Transfer in Photosystem I: Femtosecond Spectroscopy upon Excitation of Reaction Center in the Far-Red Edge of the Q Y Band. Biochim. Biophys. Acta-Bioenerg..

[56] Boatman E.M., Lisensky G.C. (2005). Faster Synthesis for CdSe Quantum Dot Nanocrystals. J. Chem. Educ..

