# Supplementary material for: Ultrafast Spectroscopy of Fano-Like Resonance between Optical Phonon and Excitons in CdSe Quantum Dots: Dependence of Coherent Vibrational Wave-Packet Dynamics on Pump Fluence
Source: Nanomaterials (Basel). 2017 Nov 4;7(11):371. doi: 10.3390/nano7110371 (PMC5707588; doi:10.3390/nano7110371)
Supplement: Supplementary file 1 [file nanomaterials-07-00371-s001.pdf]

Supporting Information.

Ultrafast Spectroscopy of Fano-Like Resonance between Optical Phonon and Excitons in CdSe Quantum Dots: Dependence of Coherent Vibrational Wave-Packet Dynamics on Pump Fluence

Victor Nadtochenko [1,2,3,*], Nikolay Denisov [2], Arseniy Aybush [1], Fedor Gostev [1], Ivan Shelaev [1], Andrey Titov [1], Stanislav Umanskiy [1], and Dmitry Cherepanov [1]

[1] N.N. Semenov Institute of Chemical Physics, Russian Academy of Sciences, Kosygina st., 4, 119991 Moscow, Russia; aiboosh@gmail.com (A.A.); boatsween@yandex.ru (F.G.); shelaev@bk.ru (I.S.); scorpio60@mail.ru (A.T.); unan43@mail.ru (S.U.); tscherepanov@gmail.com (D.C.)

[2] Institute of Problem of Chemical Physics, Russian Academy of Sciences, 142432 Chernogolovka, Russia; dnn@icp.ac.ru

[3] Chemical Faculty, Moscow State University, Leninskie Gory, 119992 Moscow, Russia

* Correspondence: nadtochenko@gmail.com; Tel./Fax: +7 499 137 66 76

SI 1. Absorption and luminescence spectra of CdSe nanoparticles.

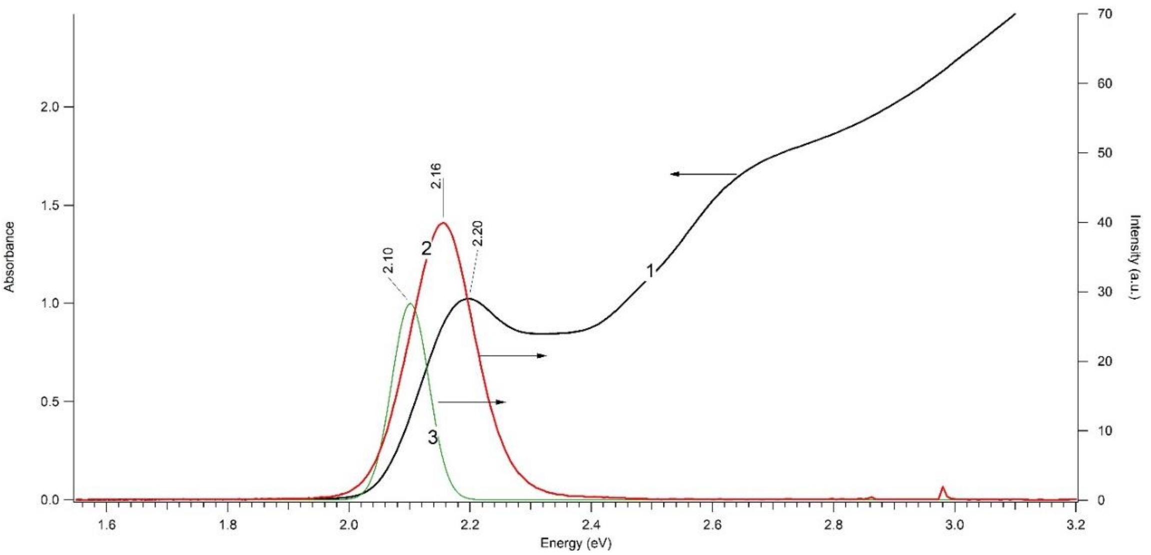

Figure S1.

1. Absorption spectrum of CdSe NP. Excitonic transition [1S(e)-1$S_{3/2}$(h)] corresponds to 573 nm (2.16 eV).

2. Luminescence spectrum of CdSe NP Excitation at 416nm (2.98 eV).

3. Gauss peak corresponding to the 30 fs femtosecond laser pulse peaked at 590 nm (2.1 eV). Bandwidth (THz): 14.7 THz. Bandwidth (cm^{-1}): 490 cm^{-1}.

Diameter of CdSe NP was estimated from the position of the lowest excitonic transition [1S(e)-1$S_{3/2}$(h)] at λ=573 nm according to the formula [1]:

$$d=59.60816-0.54736*\lambda+1.887310^{-3}*\lambda^2-2.8574310^{-6}*\lambda^3+1.6297410^{-9}*\lambda^4$$

1. Jasieniak, Jacek, Lisa Smith, Joel Van Embden, Paul Mulvaney, and Marco Califano. "Re-examination of the size-dependent absorption properties of CdSe quantum dots." *The Journal of Physical Chemistry C* 113, no. 45 (2009): 19468-19474. Doi:
2. 10.1021/jp906827m

SI 2 Transient decay at different pump fluences.

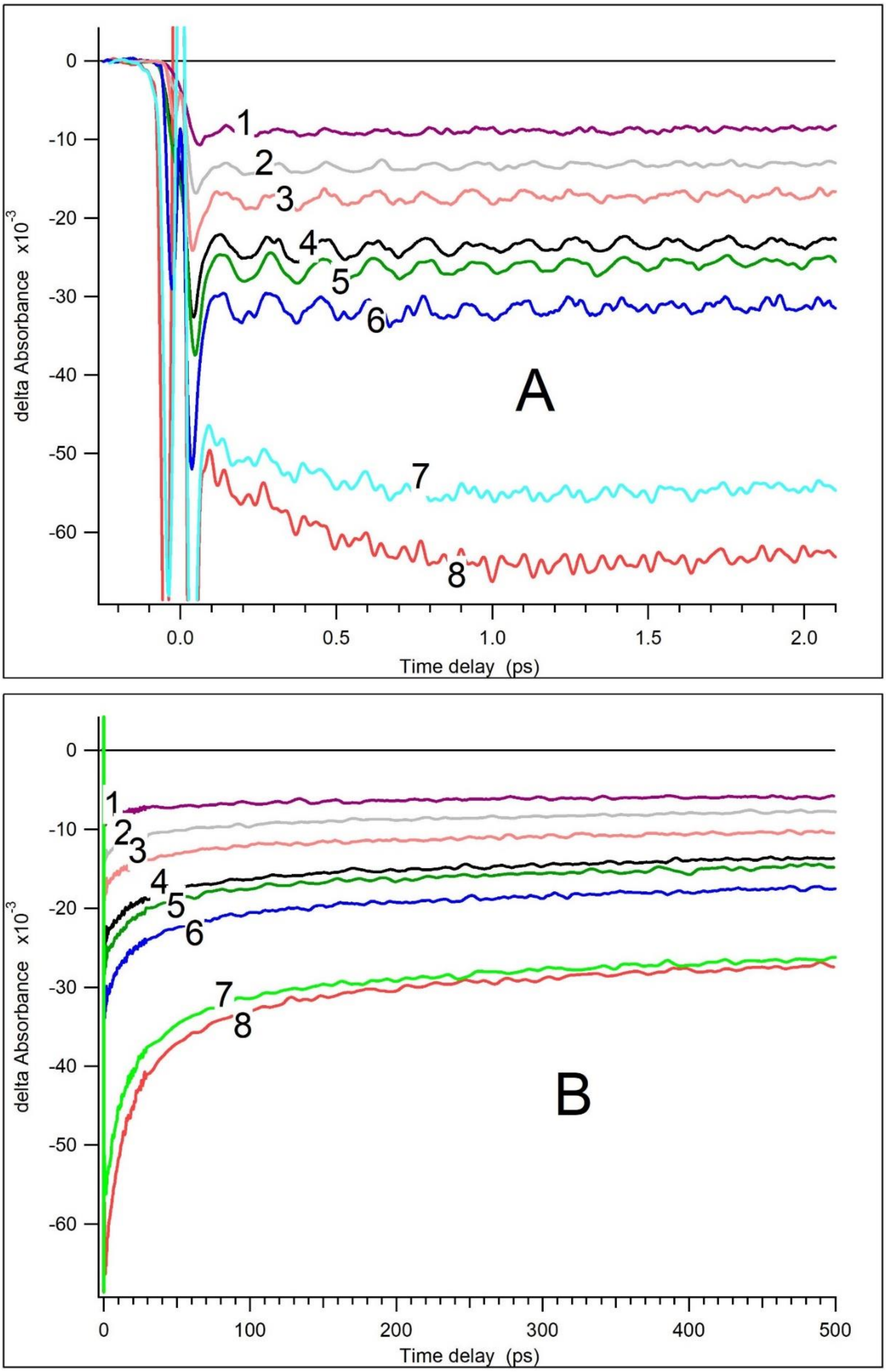

Figure S2. Transient kinetics at probe wavelength 565 nm as a function of the pump density in the time window -0.2 – 2.2 ps (**A**) and in the time window -0.2 – 500 ps (**B**): 1, 0.042 mJ/cm^2; 2, 0.064 mJ/cm^2; 3, 0.085 mJ/cm^2; 4, 0.18 mJ/cm^2; 5, 0.25 mJ/cm^2; 6, 0.35 mJ/cm^2; 7, 1.06 mJ/cm2; 8, 1.8 mJ/cm^2.

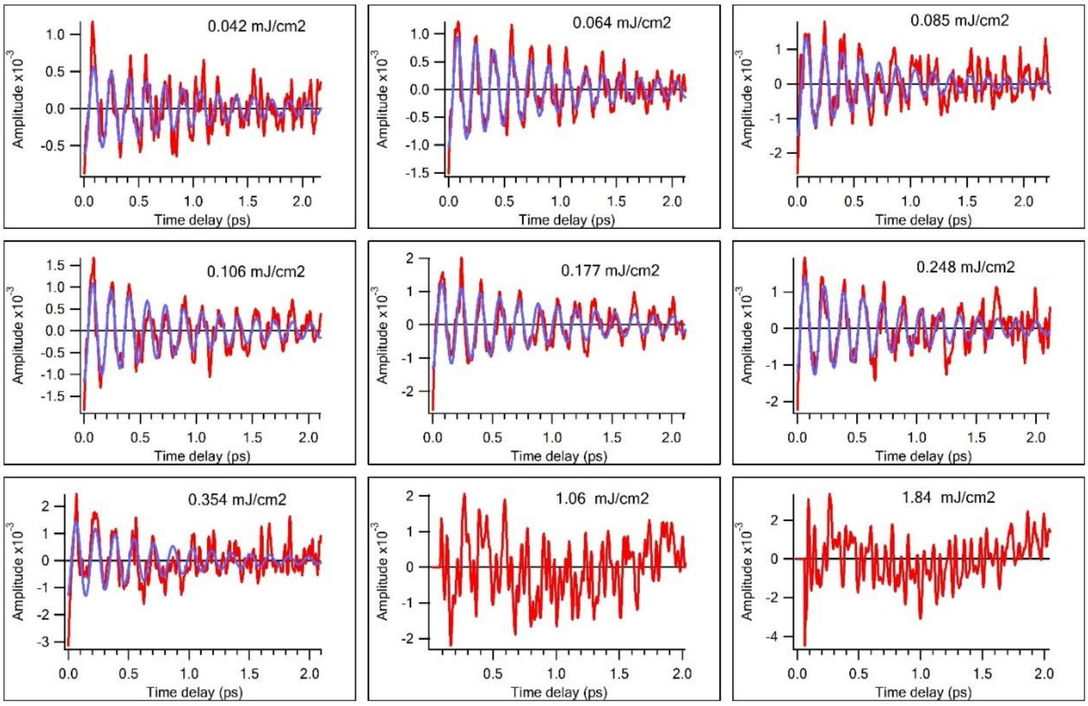

Figure S3. Residuals of transient kinetics at different level of pump energies (red curves). Blue curves are fit by dumped oscillating function A*exp(-t/τ_{damp})*sin($\omega t + \phi$). One damped sinusoid function is not enough to fit the oscillations at pump fluence higher than 1 mJ/cm^2.

SI 3 Fast Fourier Transform.

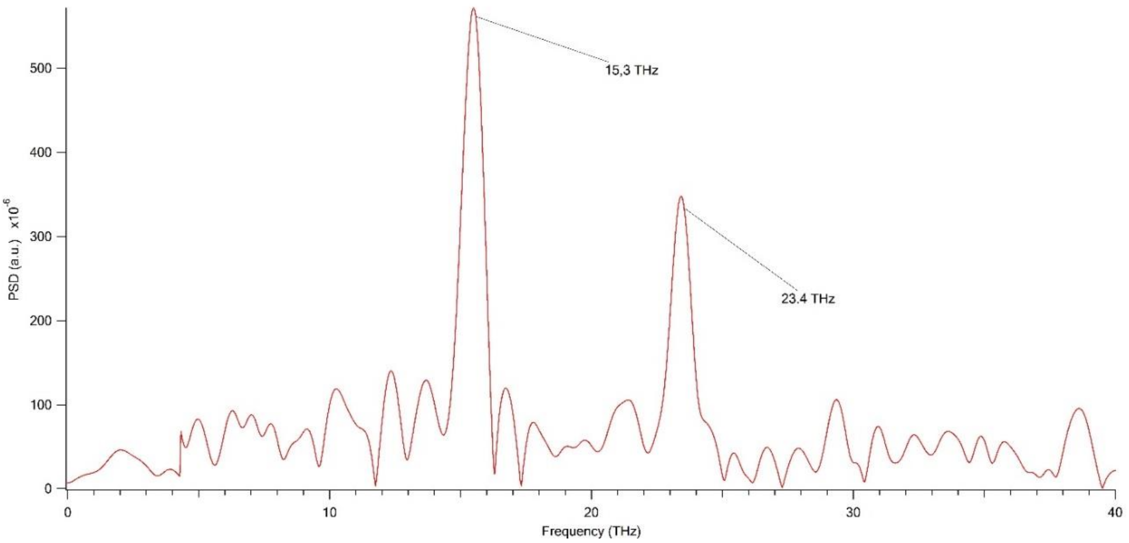

Figure S4. FFT of transients at 567 nm of pure toluene under 1.84 mJ/cm^2 pump. This spectrum evidence that peaks at 15.3 THz and 23.4 THz correspond to the wave packet generated in the toluene.

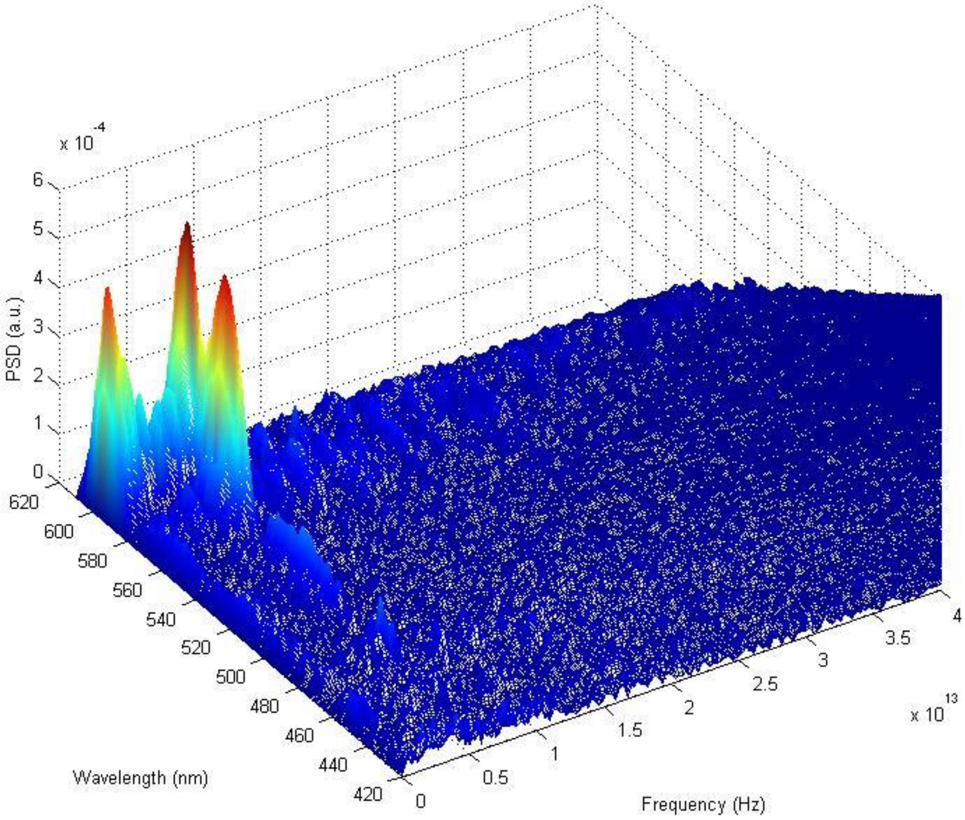

Figure S5. The FFT analysis of oscillating components in transient absorption data matrices. The pump energy density is 0.064 mJ/cm^2. The maximum PSD amplitude of LA oscillation is close to 596 nm. LO oscillation shows two PSD maximum close to 592 nm and 572 nm.

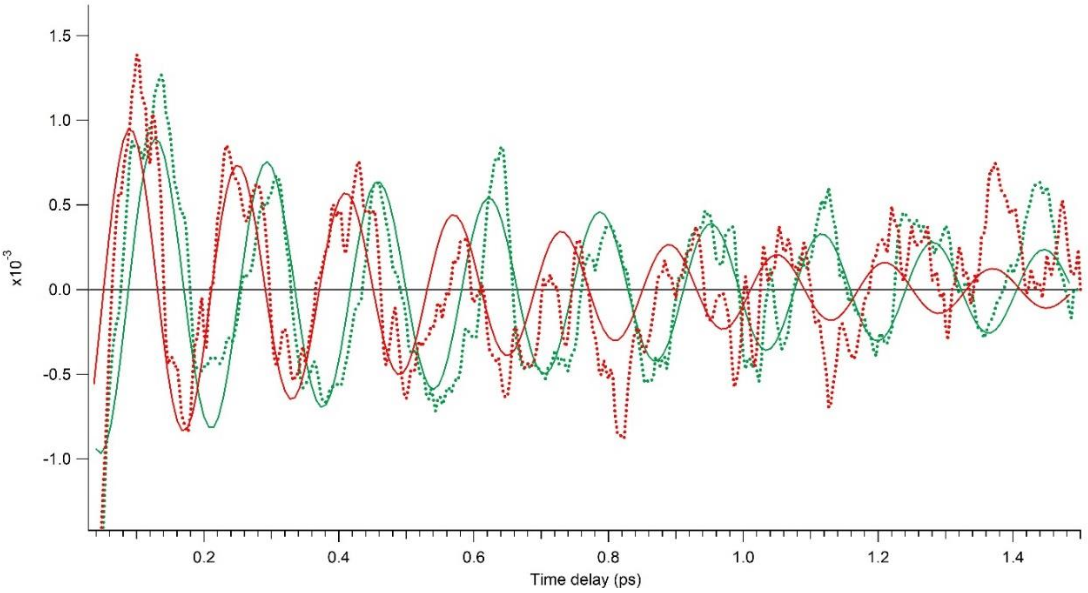

Figure S6. Oscillation in residuals of transient traces at probe wavelengths of 2.087 eV (594 nm) and 2.186 eV (567 nm). Green line corresponds to 2.186 eV (567 nm). Red line correspond to 2.087 eV (594 nm).

Parameters for fit function A*exp(-t/τ_{damp})*sin(ωt +ϕ):

1. Green line (567 nm), *A*= 0.001 ± 0.002 eV, ω= 38.15 ± 0.011 (6.0716 THz), φ=0.21, 1/τ_{damp}=1.0 ± 0.12 ps^{-1};

2. Red line (567 nm), *A*= 0.001 ± 6.28 10^{-5}eV, ω= 39.13 ± 0.012 (6.2276 THz), φ=3.99, 1/τ_{damp}=1.43 ± 0.13 ps^{-1};

The phase uncertainty is given by the timing uncertainty of 3.33 fs, corresponding to π/11.5 in the phase angle.

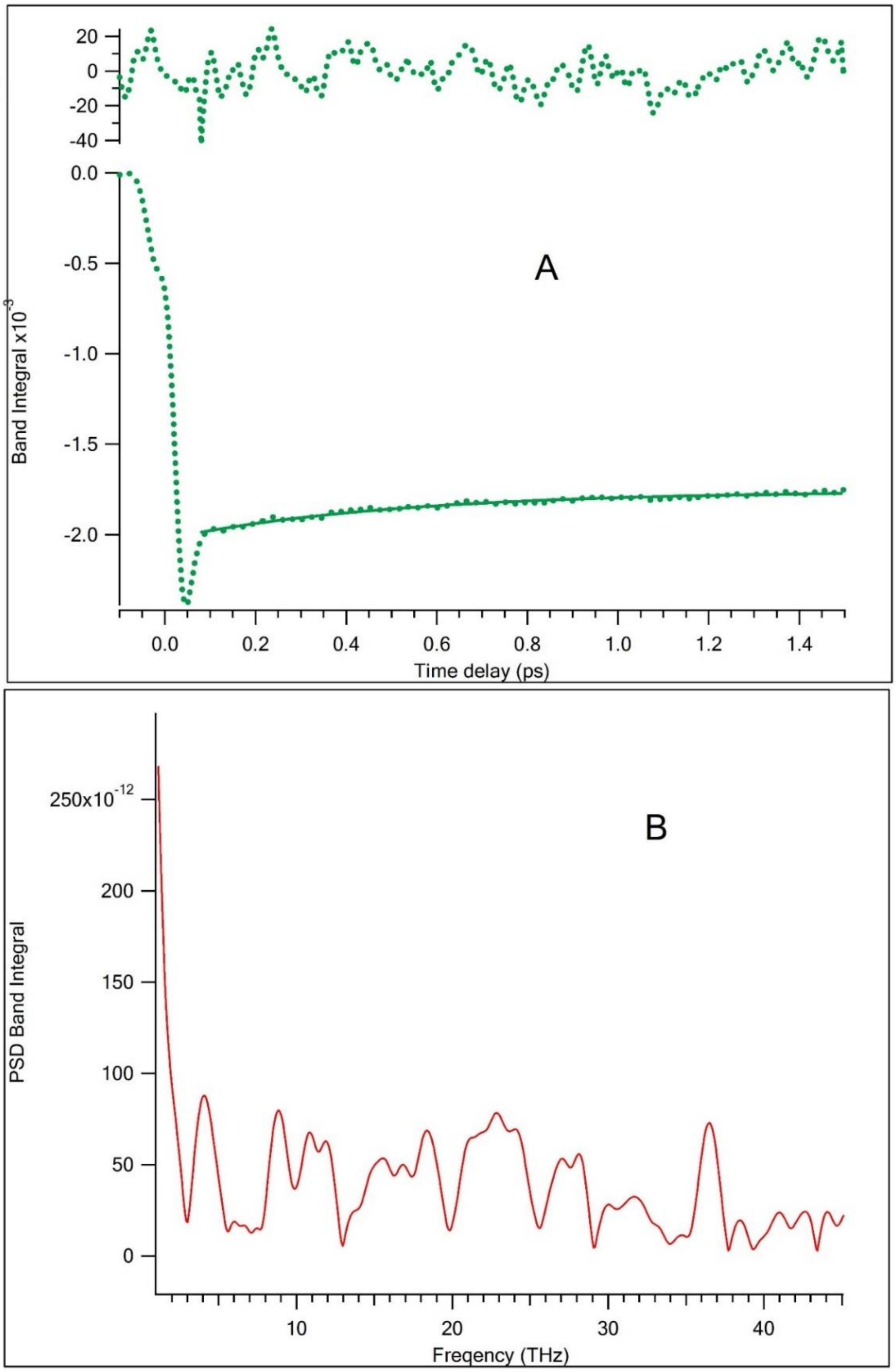

Figure S7. A. Kinetics of band integral calculated in the region of 2–2.41 eV. B. Power spectral density of the band integral residuals. The pump energy density was 0.064 mJ/cm^2. The oscillations are not observed.

SI 4. CWT of transients at 567 nm.

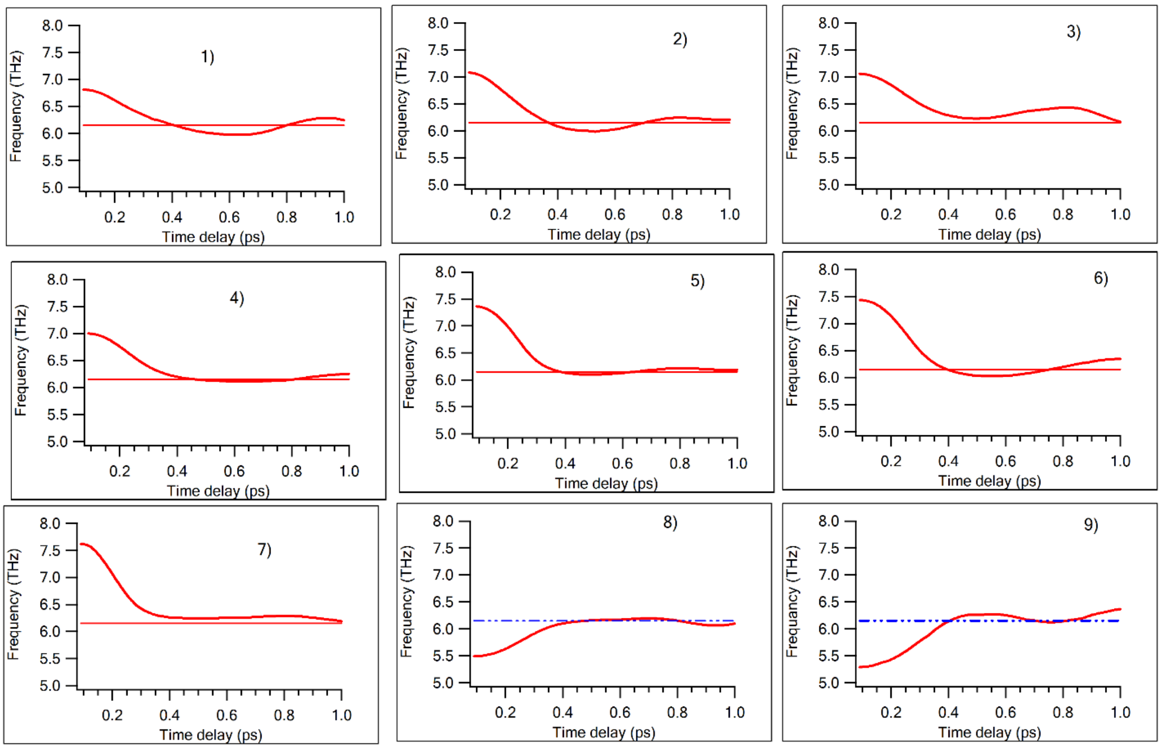

Figure S8. Changes of frequency corresponding to the maximum of CWT intensity in time under different pump fluence (data from Figure 5. 1) 0.0424 mJ/cm²; 2) 0.064 mJ/cm²; 3) 0.085 0.0424 mJ/cm²; 4) 0.106 mJ/cm²; 5) 0.18 mJ/cm²; 6) 0.25 mJ/cm²; 7) 0.35 mJ/cm²; 8) 1.06 mJ/cm²; 9) 1.84 mJ/cm²

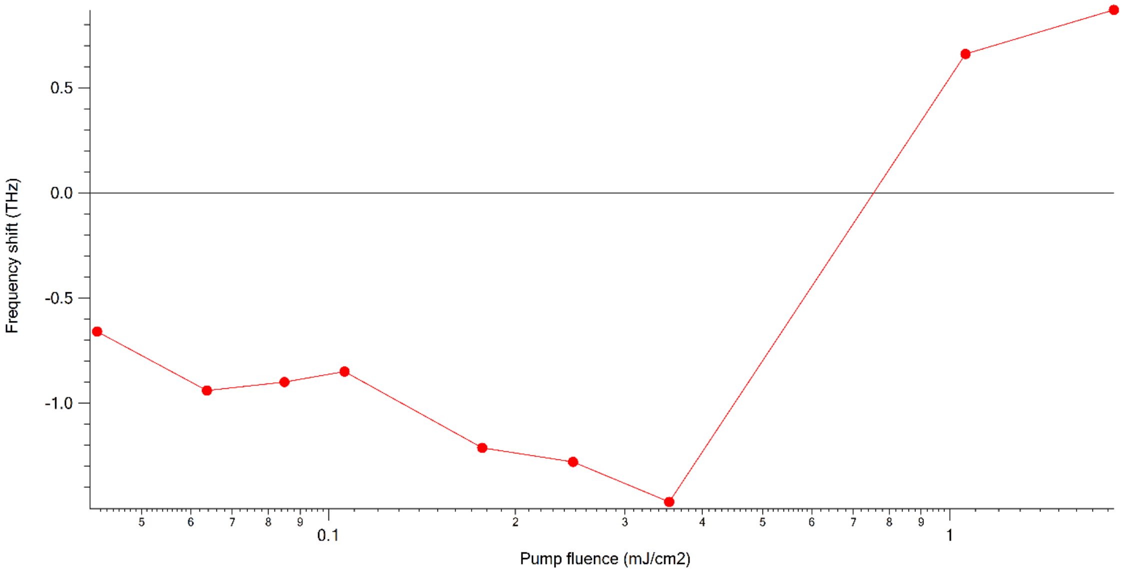

Figure S9. Frequency shift vs pump fluence. Calculated from data in Figure S8 as difference between the initial time delay of 120 fs and frequency of LO phonon of 6.16 THz revealed by FFT.

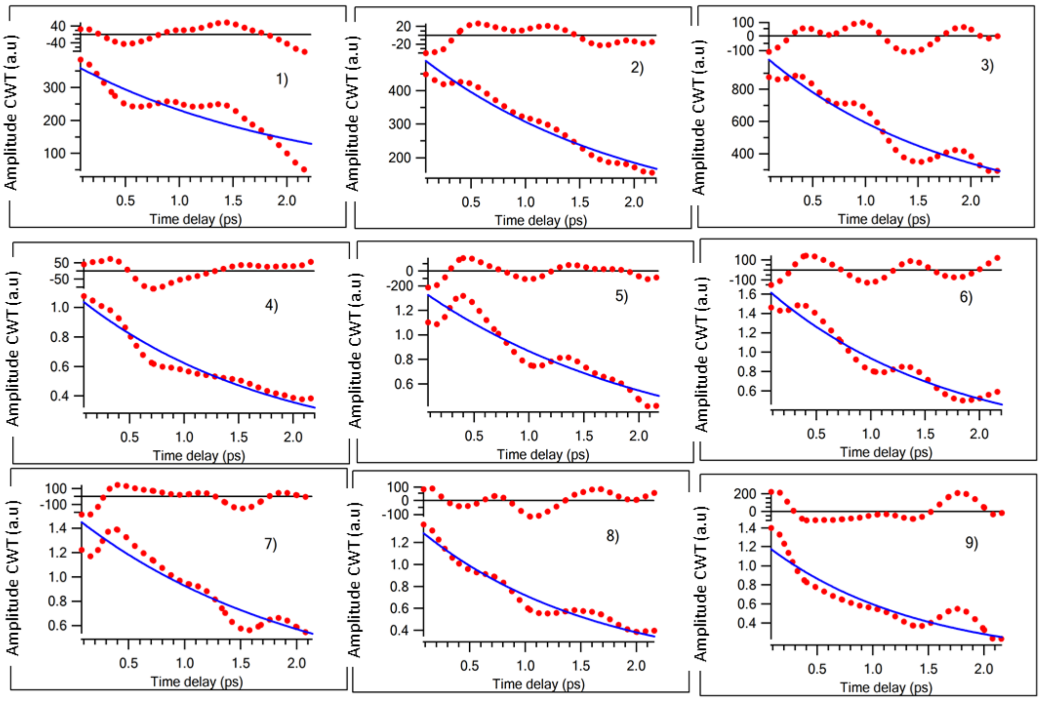

Figure S10. Changes of maximum of CWT intensity in time at different pump fluences (taken from Figure 5. 1) 0.0424 mJ/cm²; 2) 0.064 mJ/cm²; 3) 0.085 0.0424 mJ/cm²; 4) 0.106 mJ/cm²; 5) 0.18 mJ/cm²; 6) 0.25 mJ/cm²; 7) 0.35 mJ/cm²; 8) 1.06 mJ/cm²; 9) 1.84 mJ/cm².

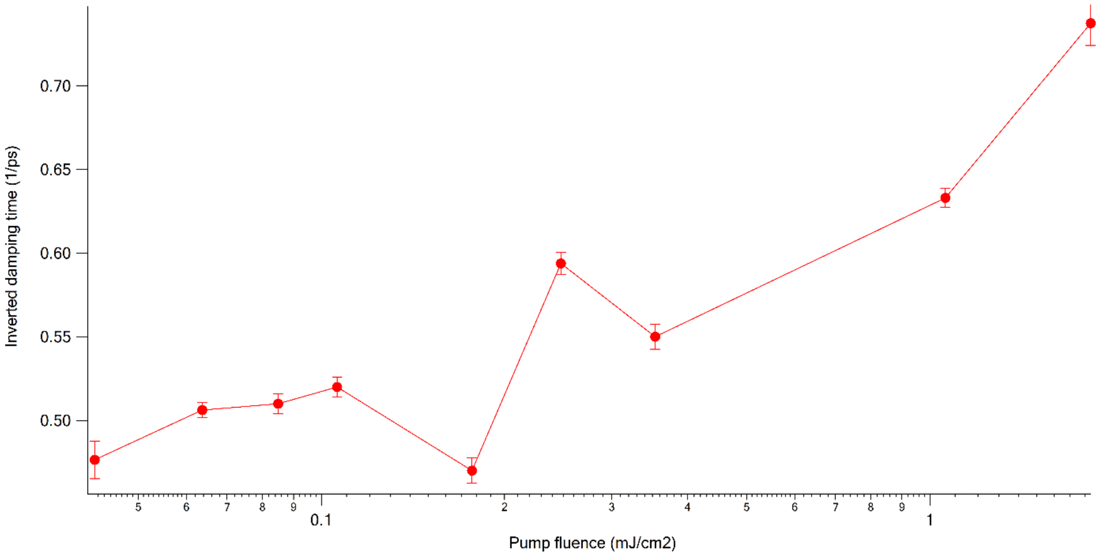

Figure S11. Dependence of the inverted damping time 1/τ on the pump fluence. Points were calculated from data of Figure SI 10.

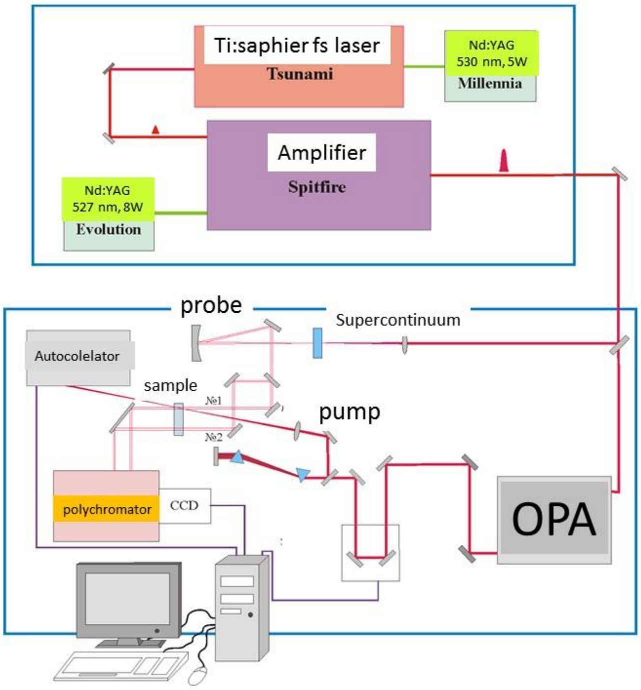

Figure S12. The femtosecond pump to supercontinuum probe setup was used to measure TA spectra. The output of a Ti:sapphire oscillator (800 nm, 80 MHz, 80 fs, «Tsunami», «Spectra-Physics», Santa Clara, CA, USA) was amplified by a regenerative amplifier system («Spitfire», «Spectra-Physics», Santa Clara, CA, USA).
